# Return to Work after a Stroke in Working Age Persons; A Six-Year Follow Up

**DOI:** 10.1371/journal.pone.0169759

**Published:** 2017-01-06

**Authors:** Emma Westerlind, Hanna C. Persson, Katharina S. Sunnerhagen

**Affiliations:** Department of Clinical Neuroscience and Rehabilitation, Institute of Neuroscience and Physiology, Sahlgrenska Academy, University of Gothenburg, Gothenburg, Sweden; University of Miami School of Medicine, UNITED STATES

## Abstract

**Objectives:**

Stroke is one of the most common and resource intensive diseases for society. Stroke in the working age population is increasing in different parts of the world. An incomplete return to work (RTW) after sick leave post stroke entails negative consequences for the affected person and an economical burden for society. The aim of this study was to explore the RTW rate and factors associated with RTW in a six-year follow up post stroke.

**Methods:**

Data from 174 persons 63 years or younger, with first ever stroke in 2009–2010 in Gothenburg were analyzed. Baseline characteristics were collected through medical records and the Swedish Health Insurance Office provided information on sick leave up to 6 years post stroke. Time-to-event was presented and cox regression as well as logistic regression were used to analyze risk factors for no-RTW.

**Results:**

The RTW rate was 74.7%, at the end of follow up. Participants continued to RTW until just over 3 years post stroke. Dependency at discharge (in the modified Rankin Scale) and sick leave prior to the stroke were significant risk factors for no-RTW after 1 year with odds ratio 4.595 and 3.585, respectively. The same factors were significant in time-to-event within six years post stroke with hazard ratio 2.651 and 1.929, respectively.

**Conclusions:**

RTW after a stroke is incomplete, however RTW is possible over a longer period of time than previously thought. More severe disability at discharge from hospital and sick leave prior to the stroke were shown to be risk factors for no-RTW. This knowledge can contribute to more individualized vocational rehabilitation.

## Introduction

Stroke is one of the most common and resource-intensive diseases with nearly 17 million events globally in 2010 [1]. Globally approximately 31% are younger than 65 years old at time of their stroke, in Sweden this percentage is 20% [2]. The total incidence of first time stroke in Sweden has decreased, however stroke in this young age group has increased [3].

Work is a wide concept that could be defined solely as paid work (employed or self-employed), or includes other occupations such as voluntary work and household work. Not working may also be divided into numerous subgroups: students, retirees, unemployed and job seeking or unemployed but not available for work [4]. It has been suggested that work is beneficial for health [5, 6] and not working could be a risk factor for stroke [7, 8]. Furthermore, working after a stroke is a significant factor in life satisfaction as a whole in younger persons, especially men who had a 2.5 times higher risk of not being satisfied if they did not return to work (RTW) [9]. Not working may not only entail isolation and reduced quality of life for the affected person, but is also a problem for governments and employers, not least economically [10]. In 2014, indirect factors (sick absence, early retirement and production years lost because of death in stroke) accounted for 21% of the total cost of stroke in Sweden [11]. Absence due to sickness is an important public health issue requiring further investigation [12].

The reported RTW rate after a stroke varies widely between different studies. An Australian study [13] showed a RTW rate of 75% within the first year after stroke, while 48% had returned to the same level of work as before the stroke in a 6 year follow up of participation in everyday activities, conducted in Sweden [14]. Further, in a one-year follow-up study, 45% had returned to paid work (30% in the same extent as before the stroke and 15% less than before) [15]. All the above studies used self-reported RTW as the outcome. A Danish study published in 2016 showed that 50% had left the workforce permanently or were still on sick-leave one year after stroke [16]. Self-rated health was strongly associated with RTW and those who had a mild or moderate stroke were significantly more likely to RTW than those with a severe stroke [16].

The aim of this study was to explore the RTW rate and factors associated with RTW in a six-year follow up post stroke.

## Materials and Methods

### Subjects and settings

All patients 18–63 years old with first ever clinical stroke with the International Classification of Diseases codes I61 intracerebral hemorrhage (ICH) or I63 ischemic stroke (IS), at the Sahlgrenska University Hospital, Sahlgrenska, Gothenburg, Sweden between February 4^th^ 2009 –December 2^nd^ 2010 were identified. Additionally, patients had to live within 35 km from the hospital and be treated at the stroke unit, neurosurgical unit or intensive care unit to be included. Sahlgrenska is the largest of three main hospitals in Gothenburg. The included patients are from the extended Stroke Arm Longitudinal Study at the University of Gothenburg (SALGOT) [17, 18].

The baseline characteristics, stroke type, sex, age, reperfusion treatment (thrombolysis and thrombectomy) and comorbidity were collected through medical charts. The National Institutes of Health Stroke Scale (NIHSS, 0–42 points, lower score is better [19]) and Glasgow Coma Scale (GCS, 3–15, higher score is better [20]) were used to measure the severity of the stroke at the time of arrival at hospital. GCS was only assessed in those patients where NIHSS was not measured; i.e. the patient with severely decreased consciousness. The patient’s disability at discharge from hospital was assessed with the modified Rankin Scale (mRS, 0–6, lower score is better [21]).

Health-related quality of life was measured with the EuroQol-5dimentions (EQ-5D) including mobility, self-care, usual activities, pain/discomfort and anxiety/depression and the additional visual analogue scale (VAS) [22]. The EQ-5D was sent out to all patients as a follow up mail survey approximately five years post stroke (median time 59 months). The mortality rate and cause of death within the study period was collected from the mortality register at the National Board of Health and Welfare in Sweden. The Swedish Health Insurance Office provided information about early retirement (sickness compensation) and sick leave (sickness benefit) one year prior to the stroke as well as the number of days of early retirement or sick leave up to six years post stroke. Due to administrative reasons the follow up questionnaire and data collection from the Health Insurance Office was not conducted at the exact same time.

### Working status

During the first two weeks of sick leave in Sweden, the employer provides sick pay. Thereafter the Swedish Health Insurance Office, which is a public authority, pays the sickness benefit. In the RTW process, the Health Insurance Office uses a step-by-step rehabilitation model to evaluate a person’s capacity to work with more stringent criteria being applicable for a longer absence from work. Employers are eligible to wage subsidies if they hire people with a disability [10]. Early retirement is a potential arrangement instead of sick leave for a person where RTW is not likely due to disease, however RTW is still possible even after early retirement. The participants that were in early retirement prior to the stroke were not included in the main analyses in the current study. The participants’ entry into the study began at admittance to the hospital with stroke symptoms. The study period was defined as number of days registered with sickness benefit or sickness compensation post stroke. The main outcome measure was RTW. RTW was defined as no longer being registered on sickness compensation or sickness benefit (neither full time nor part time) in the Swedish Health Insurance Office and not leaving it within a year from turning 65 years old (old-age retirement in Sweden) or within a month from dying.

### Covariates

Potential predictor variables for RTW were disability at discharge from hospital (mRS score) and sick leave prior to the stroke. The mRS was dichotomized into functional independency if scores were between 0–2, and scores 3–6 represented dependency [23]. Sick leave prior to the stroke included those who received sickness benefit for at least two weeks in the last year before their stroke. Age, sex and stroke type were handled as confounders and were adjusted for in the multiple regression models.

Comorbidity was presented as no, mild, moderate or severe comorbidity according to the Charlson Comorbidity Index [24], but also the presence of comorbidity potentially affecting RTW. The last was counted as yes if the participant had one of the following diseases: Myocardial infarction, diabetes, alcohol/drug abuse, mental illness, cancer, chronic obstructive pulmonary disease, rheumatic disease or other specific diseases.

NIHSS was presented with the total score and items that represented cognitive function (Cog4, 0–9 points [25]) and motor function (NIHSSmotor 0–16 points) were also handled as separate variables. GCS was presented in characteristics as mild (13–15), moderate (9–12) or severe (3–8) where NIHSS data were missing [26].

Answers from the EQ-5D questionnaire were recalculated into a single index value that, like the VAS, represents health-related quality of life. The index was calculated for each participant using a Swedish tariff [27].

### Statistical methods

The data was processed and analyzed in IBM SPSS 22. The level of significance was set as p-value < 0.05. Fishers Exact test and Mann Whitney U test were used to investigate demographic differences between groups.

Non-parametric comparisons of time to RTW, up to six years post stroke, between the two stroke-types were made by Kaplan-Meier curve and the log rank test [28]. One cause of censoring was death during the study period. This censoring is in violation of the non-informative-censoring-assumption. In order to handle some of the consequences of the violation, death before RTW was treated with a worse case scenario approach. It means that participants that died before RTW were set to censoring at the end of the study period instead of the classical date of death. This is done in order to get a more conservative estimate for RTW. Another cause of censoring is old-age retirement and this is set at time of retirement since it is not in violation of the non-informative-censoring-assumption.

Semi-parametric analysis [28] of time to RTW was made using a multiple cox proportional hazard model, based on data from the whole study period and the estimates of the predictors were presented as association with no-RTW. The variables in the model were included based on clinical and theoretical relevance.

The risk factors for no-RTW within one year post stroke were investigated using logistic regression [28], including the same predictors and confounders as in the prespecified cox model. The outcome of the logistic model was set to no-RTW within one year post stroke. Age-stratified logistic regression models for the odds of no-RTW one year post stroke were also performed in order to investigate possible differences in risk factors between age groups. Strata were constructed using the age 50 years old at stroke onset as the cut off, creating a younger and an older group.

To test the goodness of fit of the logistic regression models, Hosmer and Lemeshow tests and Receiver Operating Characteristics curves (ROC-curves) were performed. For the cox model, Kaplan-Meier curves and log(-log(survival curves)) were used to check for serious violations against the proportional hazard assumption. Graphical representation of partial residuals versus survival time was used to check for time-dependent covariates. If suspected time-dependence, a further cox regression model with a time-dependent variable to assess potential significant time-dependence was used.

### Ethical considerations

The study followed the Helsinki declaration and was approved by the Regional Ethics Committee in Gothenburg (EPN) in May 5^th^ 2008 (Dnr: 225–08) with an additional application T801-10. The follow up questionnaires were approved in June 5^th^ 2013 (Dnr: 400–13) and an amendment for the Health Insurance Office was approved on October 22^nd^ 2015 (Dnr T830-15). Written informed consent was provided by the subjects to participate in the questionnaire follow-up part of this study. In Sweden, data that are handled within the frame of national quality registers is an exception from the general rule of patient consent because it provides improvement of the quality of care and treatment that is of general interest. This have been decided by the Swedish Data Inspection Board. Further, according to Swedish law on personal particulars data (personuppgiftslagen, Swedish law No. SFS 1998:204) no informed consent is needed to collect data from medical charts for clinical purposes and quality control.

## Results

### Characteristics

The total number of patients 18–63 years old at stroke onset was 211, whereof the majority were men and the prevalence of ischemic stroke were 78%, see Table 1. The median age at time of the stroke was 53 years old.

**Table 1:** Baseline characteristics of the study population.

Characteristics	Participants
**Total,** *n*	211
**Age at stroke**, years *median (min-max)*	53 (21–63)
≤ 50 years old, *n (%)*	87 (41.2)
≥ 51 years old, *n (%)*	124 (58.8)
**Sex**, *n (%)*	
Female	69 (32.7)
Male	142 (67.3)
**Stroke type**, *n (%)*	
IS	164 (77.7)
ICH	47 (22.3)
**Reperfusion treatment** (IS, n = 164), *n (%)*	
Yes	22 (13.4)
No	142 (86.6)
**Health insurance prior to stroke**, *n (%)*	
Early retirement	37 (17.5)
Sick leave	26 (12.3)
**Charlson Comorbidity Index,** *n (%)*	
No comorbidity	149 (70.6)
Mild comorbidity	54 (25.6)
Moderate Comorbidity	5 (2.4)
Severe comorbidity	3 (1.4)
**Comorbidity affecting RTW,** *n (%)*	
Myocardial infarction	6 (2.8)
Diabetes	11 (5.2)
Alcohol/Substance abuse	13 (6.2)
Mental illness	16 (7.6)
Cancer	6 (2.8)
Chronic obstructive pulmonary disease	5 (2.4)
Rheumatic disease	3 (1.4)
Other	11 (5.2)
**NIHSS**^a^	
*Median (min-max)*	1 (0–22)
*Mean (SD)*	3.6 (5.1)
**NIHSSmotor**^a^	
*Median (min-max)*	0 (0–8)
*Mean (SD)*	1.3 (2.3)
**Cog4**^a^	
*Median (min-max)*	0 (0–8)
*Mean (SD)*	0.4 (1.1)
**GCS**^b^, *n (%)*	
Mild (13–15)	0 (0.0)
Moderate (7–12)	1 (9.1)
Severe (3–6)	10 (90.9)
**mRS**, *n (%)*	
Independent (0–2)	107 (50.7)
Dependent (3–6)	104 (49.3)
*Median (min-max)*	2 (0–6)

^a^ n = 200.

^b^ n = 11.

Abbreviations: IS: ischemic stroke; ICH: intracerebral hemorrhage; NIHSS: National Institutes of Health Stroke Scale; GCS: Glasgow Coma Scale; mRS: modified Rankin Scale; SD: standard deviation.

**Fig 1:**
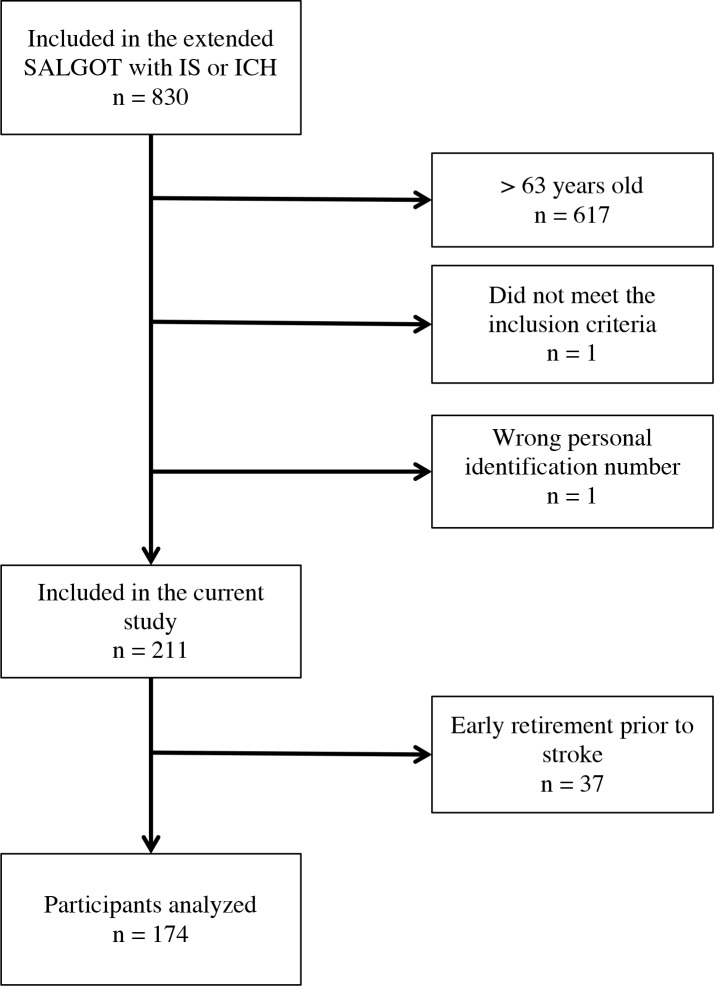
Flowchart of the study population. Abbreviations: SALGOT: the Stroke Arm Longitudinal Study at the University of Gothenburg; IS: ischemic stroke; ICH: intracerebral hemorrhage.

Of the 211 persons, 26 (12.3%) were on sick leave and 37 (17.5%) had early retired prior to the stroke (Fig 1). The most common group of diseases among the early retired persons were mental illness followed by pain and cardiovascular disease. Women were predominant in the early retirement group (p 0.033), and were also older than the working participants (p 0.011). There were no significant differences in stroke type, NIHSS or mRS. When comparing the groups on sick leave or not prior to the stroke, there were no significant differences regarding sex, age, stroke type, NIHSS or mRS.

Nineteen persons had died (7 within the first week after stroke onset) up until the mailed survey five years post stroke. Of the 19, 7 were in early retirement prior to the stroke, 10 died before RTW and 2 after RTW.

### RTW

After excluding the 37 persons in early retirement prior to the stroke, 174 persons remained (Fig 1). Out of these, 130 (74.7%) did RTW, 10 (5.7%) went into old-age retirement, 10 (5.7%) deceased before RTW and 24 (13.8%) were still on sickness compensation six years after their stroke. No participants remained on sickness benefit until end of follow up and no participants that received sickness compensation did RTW.

**Fig 2:**
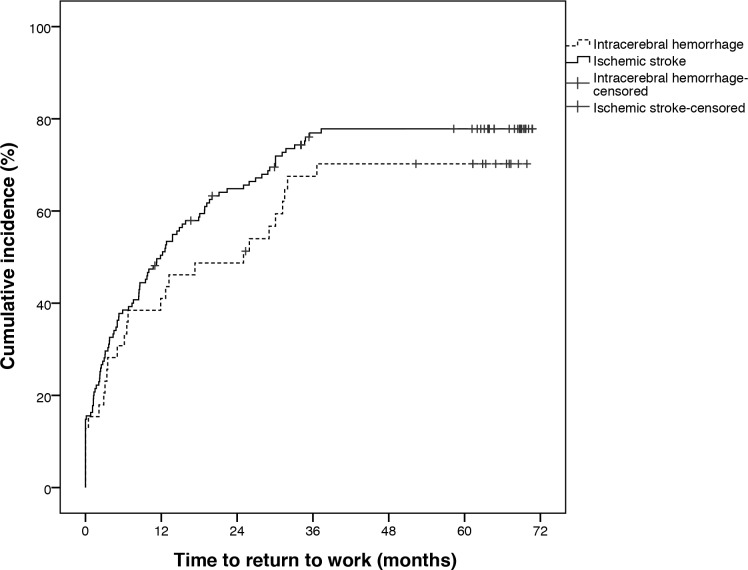
Cumulative incidence in time to RTW in months post stroke, divided according to stroke type.

The time to RTW, divided into stroke type, is shown in Fig 2. The participants with IS returned at a slightly higher rate than those with ICH, however the difference was not significant. The first quartile RTW within 2.7 months after stroke (S.E. 0.7) and the second quartile RTW within 12.7 months (S.E. 2.2). The curve plateaued for RTW at just over 3 years. After the first year post stroke, 84 persons (48.3%) had RTW.

### Factors associated with RTW

Thirty three (19.0%) of the 174 persons had a comorbidity that potentially could affect the RTW, however as seen in Table 2 there was no significant difference in RTW between the persons with comorbidity and the persons without (p 0.506). The NIHSS score for cognitive function (Cog4) and motor function (NIHSSmotor) were assessed in 166 participants (95%). There were significant differences between the RTW and no-RTW groups at six years in both cognition (p < 0.0005) and motor function (p < 0.0005) with less impairment in the RTW group.

**Table 2:** Comparison of factors between RTW and no-RTW groups.

Factors	RTW	No-RTW	p-value
**Total**, *n*	130	44	
**Age**, years *median (min-max)*	51 (21–63)	57 (33–63)	0.006
**Sex,** *n (%)*			0.704
Female	37 (28.5)	14 (31.8)	
Male	93 (71.5)	30 (68.2)	
**Stroke-type,** *n (%)*			0.405
IS	103 (79.2)	32 (72.7)	
ICH	27 (20.8)	12 (27.3)	
**Sick-leave prior to stroke,** *n (%)*			0.232
Yes	17 (13.1)	9 (20.5)	
No	113 (86.9)	35 (79.5)	
**Comorbidity affecting RTW,** *n (%)*			0.506
Yes	23 (17.7)	10 (22.7)	
No	107 (82.3)	34 (77.3)	
**NIHSS**^**a**^,			< 0.0005
*Median (min-max)*	1 (0–22)	3 (0–18)	
*Mean (SD)*	2.8 (4.5)	5.8 (6.1)	
**NIHSSmotor**^**a**^,			< 0.0005
*Median (min-max)*	0 (0–8)	1 (0–8)	
*Mean (SD)*	1.1 (2.2)	2.3 (2.8)	
**Cog4**^**a**^,			< 0.0005
*Median (min-max)*	0 (0–8)	0 (0–6)	
*Mean (SD)*	0.2 (0.8)	1.0 (1.7)	
**mRS,** *n (%)*			< 0.0005
Independent	80 (61.5)	10 (22.7)	
Dependent	50 (38.5)	34 (77.3)	

^a^ n = 166.

Abbreviations: RTW: return to work; IS: ischemic stroke; ICH: intracerebral hemorrhage; NIHSS: National Institutes of Health Stroke Scale; mRS: modified Rankin Scale; SD: standard deviation.

In the logistic regression at one year post stroke shown in Table 3, mRS and sick leave were incorporated in the multivariable model and adjusted for age, sex and stroke type. Being dependent in mRS and being on sick leave prior to the stroke were significantly associated with no-RTW with OR 4.595 and OR 3.585, respectively.

**Table 3:** Adjusted estimates in logistic regression of factors associated with no-RTW one year post stroke.

Predictors	OR	95% CI	p-value
Sick-leave prior to stroke	3.585	1.315–9.774	0.013
Dependent in mRS	4.595	2.333–9.051	< 0.0005

N = 174. Hosmer and Lemeshow test, p = 0.540. ROC-curve, area under the curve = 0.735. Abbreviations: OR: odds ratio; CI: confidence interval; mRS: modified Rankin Scale.

In the cox regression at six years post stroke (Table 4), mRS and sick leave were incorporated in the multivariable model and adjusted for age, sex and stroke type. Being dependent in mRS and being on sick leave prior to the stroke were significantly associated with no-RTW with HR 2.651 and 1.929, respectively.

**Table 4:** Adjusted estimates in cox regression of factors associated with no-RTW up to six years post stroke.

Predictors	HR	95% CI	p-value
Sick-leave prior to stroke	1.929	1.149–3.240	0.013
Dependent in mRS	2.651	1.831–3.837	< 0.0005

N = 174. No apparent disproportionality and no significant time-dependence were found in the covariates. Abbreviations: OR: odds ratio; CI: confidence interval; mRS: modified Rankin Scale.

### Age divided analyses

There was no significant difference in RTW rate within the first year after stroke between the younger (≤ 50 years old) and the older group (≥ 51 years old). As seen in Table 5, being dependent in mRS proved to be significantly associated with no-RTW with OR 6.690 in the older group. In the younger group no significance was found. Sick leave prior to stroke did not meet the size criteria of logistic regression and hence were not included in the analyses.

**Table 5:** Adjusted estimates in logistic regression of factors associated with no-RTW one year post stroke in age divided groups.

Predictors	OR	95% CI	p-value
**Older group** (≥ 51 years old)			
Dependent in mRS	6.690	2.655–16.858	< 0.0005
**Younger group** (≤ 50 years old)			
Dependent in mRS	2.215	0.876–5.601	0.093

Older group: n = 97. Hosmer and Lemeshow test, p = 0.393. ROC-curve, area under the curve = 0.740. Younger group: n = 77. Hosmer and Lemeshow, p = 0.891. ROC-curve, area under the curve = 0.600. Abbreviations: OR: odds ratio; CI: confidence interval; mRS: modified Rankin Scale.

### Health-related quality of life

Seventy-six (46.9%) out of the 162 surviving participants answered EQ-5D. There was no significant difference between responders and non-responders regarding age (p 0.380), sex (p 0.494), stroke type (p 0.850), NIHSS (p 0.537), mRS (p 0.875) or sick leave prior to the stroke (p 1.000).

There was no significant difference in the index value calculated from EQ-5D, representing health-related quality of life, between the RTW and no-RTW groups (p 0.061) after six years. In contrast, the difference between the groups in the VAS was significant (p 0.012) with a higher self-rated health-related quality of life in the RTW group.

## Discussion

In this six-year follow up after stroke, 74.7% of the participants did RTW. A more severe disability at discharge was a significant factor for no-RTW after one and six years. Being on sick leave prior to the stroke was also a significant risk factor for no-RTW at one and six years post stroke.

In the current study participants continued to RTW even up to three years post stroke, not shown previously with shorter follow-up time [13, 16]. The finding is important not only for patients but also for governments when creating policies regarding this major health and economic issue. Knowledge surrounding late RTW could improve our understanding of the time and patience these patients need with vocational rehabilitation and avoid put unreasonable pressure on the patients to RTW in the fastest possible way. In the no-RTW group, the most common outcome was early retirement. Even though the system of entering early retirement instead of sick leave after a long-term absence from work might reduce the RTW rate in Sweden, the system of giving wage subsides to employers for hiring persons with a disability could possibly contribute to a higher RTW rate. In the current study, the RTW rate at one year was 48.3%, compared with 75% in an Australian study [13], 50% in a Danish study [16], 62.4% in a Japanese study [29] and 35% in a study conducted in the UK [30]. Three different reviews with varying follow up time, mostly shorter than six years, additionally present substantially lower RTW rate than in the current study [31–33]. Due to different sick-leave systems, socio-political contexts and cultural differences the RTW after sickness may vary between countries [34]. The current study was set in a University Hospital with specialized treatment methods available. Furthermore, Sweden is a high-income country with tax funded care and rehabilitation and this could potentially affect the generalizability of the results to other health care systems [35]. Furthermore, the study design with a six-year follow up and the use of national records instead of self-reported measurements to assess RTW, reduced the risk of drop out and recall bias and could also partly explain the differences between studies.

Functional independency (low mRS score) at discharge from hospital has shown to be a significant factor for RTW both in the current study and previous [36–38]. The current results furthermore indicate that being dependent at discharge (mRS score) is a stronger risk factor for no-RTW in the older patients with an OR of 6.690, while in the younger group it was not a significant factor. The current study chose not to incorporate NIHSS at arrival to hospital in the regression analyzes since it may change significantly from stroke onset to post treatment [39]. A review suggests that different deficits are difficult to combine with different type of job demands [32]. For instance, cognitive impairment might be an obstacle in some types of work while motor deficits are difficult to overcome in others. Both the scores for cognitive and motor deficits in NIHSS were significantly lower in the RTW group compared to the no-RTW group. Few previous studies have incorporated sick leave prior to the stroke as a potential predictor, however it became clear in the current study that it was a significant risk factor for no-RTW. In traumatic brain injuries, sick leave prior to the event have shown to give a high risk of no-RTW afterwards [40]. It may be important to consider the heterogeneity in the prior sick leave group. Differences in length of sick leave and reasons for sick leave within the group naturally result in a different probability of RTW post stroke. Knowledge of that the functional level of dependency at discharge from hospital and sick leave prior to the stroke have an impact on RTW help to individualize vocational rehabilitation efforts.

A substantial proportion of the participants in this study were on sick leave (12.3%) or early retirement (17.5%) prior to the stroke. For comparison, 9.0% of the total population in Sweden were on sick leave (≥ 14 days) during 2010 and 4.4% were early retired (full time or part time). This may indicate that the persons who suffer a stroke have poorer health than the general population. Older women had a higher risk of being early retired prior to the stroke. Previous research has shown that unemployment [7] and accumulated sick leave were risk factors for later stroke [8]. Early retirement however was not associated with a higher stroke rate [8], contrary to what is indicated in the current study.

There was a significantly higher VAS score of health-related quality of life in the RTW group compared to the no-RTW group. No significant difference could be seen in the index value calculated from EQ-5D. The reason for this discrepancy is unclear but the relatively low response rate may have an impact. The association between high scores and RTW could mean that people who RTW experience a higher health-related quality of life, but also that less problems in the domains that EQ-5D measure could facilitate RTW. Previous research supports a higher health-related quality of life in the RTW group [9, 41], which emphasizes the importance of optimizing the system of support people RTW.

### Limitations

There are some issues with the definition of RTW that need to be discussed. The current study was not able to evaluate if the participants returned to the same work with the same amount of working hours etc. RTW was counted as no longer being registered on sickness compensation or sickness benefit and not leaving it due to old-age retirement or death. The reason for non-registration in the Health Insurance Office could however involve more than RTW. Persons with all kinds of occupations are eligible to sickness benefit from this authority. For instance, a person that is no longer granted sickness benefit or sickness compensation but does not RTW could receive social assistance from the Social Services in Sweden. This was not investigated in the current study. Further, it could be that those who are self-employed do not register at the Health Insurance Office to the same extent as employees as it is the employer that provides sick pay the first two weeks of absence. Though this would probably apply mainly to short-term sickness absence. The use of national records enabled inclusion and analysis of persons closer to retirement age since exact number of days on sick leave was registered and retirement was counted with as a reason for censoring. However, to include persons above 60 years may lead to more censoring which could affect the estimates. The main outcome did not take into consideration work stability following the RTW. Stroke sequele might be difficult to combine with work even in the long term after RTW. In a Danish two year follow up study, about 30% did make the transition from working to sick leave within one year after initial RTW post stroke [16]. A suggestion for further research is to investigate work stability after a stroke in the long-term perspective.

The population in the current study lived in or in the vicinity of Gothenburg (urban area), which may have an impact on the results. The low response rate (47%) in the follow-up EQ-5D decreases the reliability of the results from that questionnaire. A relatively modest number of possible predictors for RTW were used, resulting in the risk of important predictors and confounders being neglected. For instance, the type of occupation, educational level and comorbidity have shown to be significant factors in previous research [41–43] but have not been considered here. Furthermore, the relatively large CI could indicate that there is relatively small number of participants in each subgroup, which results in an uncertainty regarding the exact OR/HR.

## Conclusions

Not everyone RTW after a stroke. The current study has shown that RTW occurs up to over three years post stroke. Being functionally dependent at discharge from hospital as well as having sick leave prior to the stroke were risk factors for not RTW after stroke.
